# Inequality in unmet dental care needs among South Korean adults

**DOI:** 10.1186/s12903-017-0370-9

**Published:** 2017-04-26

**Authors:** Nayoung Kim, Chang-yup Kim, Hosung Shin

**Affiliations:** 1 Department of Postanesthetic Care Unit, Yonsei Cancer Center, Severance Hospital, Seoul, Korea; 2 Department of Health Policy and Management, Graduate School of Public Health, Seoul National University, 1 Gwanak-ro, Gwanak-gu, 151-742 Seoul Korea; 3 Department of Social and Humanity in Dentistry, College of Dentistry, Wonkwang University, Iksan, Jeolabuk-do Korea

**Keywords:** Inequality, Unmet dental care needs, Normative dental treatment, Self-perceived oral health status, Socioeconomic factors, Complex sampling design

## Abstract

**Background:**

The current public health research agenda was to identify the means to reduce oral health inequalities internationally. The objectives of this study were to provide evidence of inequality in unmet dental needs and to find influencing factors attributable to those among South Korean adults.

**Methods:**

Pooled cross-sectional data from the fourth Korean National Health and Nutrition Examination Survey (2007–2009) on 17,141 Korean adults were used. Demographic factors (sex, age, and marital status), socioeconomic factors (education level, employment status, and income level), need factors (normative dental needs and self-perceived oral health status), and oral health-related factors (the number of decayed teeth, the presence of periodontitis, and the number of missing teeth) were included. Multiple logistic regression analysis was performed.

**Results:**

Of South Korean adults, 43.9% had perceived unmet dental needs, with the most common reason being financial difficulties. The disparities in unmet dental care needs were strongly associated with income level, normative treatment needs, and self-perceived oral health status. The low-income group, people with normative dental treatment needs, and those with perceived poor oral health status were more likely to have unmet dental needs. There was considerable inequality in unmet dental care needs due to economic reasons according to such socioeconomic factors as income and education level.

**Conclusions:**

Public health policies with the expansion of dental insurance coverage are needed to reduce inequalities in unmet dental care needs and improve the accessibility of dental care services to vulnerable groups who are experiencing unmet dental care needs due to socioeconomic factors despite having normative and self-perceived needs for dental treatment.

## Background

Oral health is closely associated with general health and has a great influence on quality of life [1, 2]. Although the level of oral health is improving globally, the social burden remains high for oral diseases, such as dental caries, periodontal disease, and tooth loss [3]. Previous studies find that oral health problems and inequalities are influenced by demographic and socioeconomic factors, such as education, occupation, income, and the use of health care services [4–7]. The current public health research agenda is to identify the means to reduce oral health inequalities internationally with a view to ensuring that dental services meet a population’s needs [7, 8].

Since the health security system was introduced in South Korea in 1977, the accessibility of health care has been enhanced by the 1989 implementation of the National Health Insurance (NHI) system for all citizens. Although Korea’s NHI system covers most medical services for all Koreans, the dental health insurance coverage is insufficient, and many treatments fall under non-payment services. Outpatient out-of-pocket payment for medical fees in general hospitals is approximately 30% in Korea, whereas out-of-pocket payment for dental care fees in dental clinics is approximately 75%. Compared with a low minimum hourly wage of US $4.99 (5580 Korean won) in Korea, the cost of a single crown is approximately $450, dentures are approximately $1300, and the dental implant of a single tooth is approximately $1600. According to data from the Korean National Statistical Office, income inequality has persistently increased, social polarization and inequality became major issues on the policy agenda [9]. There are several studies reporting socioeconomic disparities in oral health and dental service use [10–14]. As the income, education and occupation gap between the rich and poor have widened [9], oral health inequalities have become more pronounced in Korea. Moreover, regional disparities remain prevalent in the distribution of the dental work force between metropolitan and rural areas [15]. For this reason, many people either postpone dental treatments or do not receive comprehensive dental care service, and dental care accessibility is unequal among social classes [16].

The different types of ‘need’ have been proposed on the previous psychosocial literatures, and a wide variety of definitions of ‘need’ has been developed. Maslow hierarchized the need five levels from fundamental levels of needs for physiology to the need for self-actualization [17]. Bradshaw also set out 4 types of need; normative need, felt need, expressed need, and comparative need [18]. Most of all, ‘health needs’ should include personal, social, and health care, and the definition of ‘need’ has significant values for healthcare provision [19], and the different elements of need relate to one another. Previous studies examining unmet dental needs have mainly focused on children [20–25], and high levels of unmet dental needs have been observed among children. A few studies have investigated the factors affecting professionally assessed dental treatment needs as the outcome variable [26, 27]. Most studies of unmet dental needs have also used self-perceived oral health status to replace professionally assessed dental treatment needs [28–30]. However these studies did not take into consideration professionally assessed treatment needs and were limited in that they did not consider sufficient evaluations of dental treatment needs. Although self-assessment can provide a quick overall picture of self-perceived needs, the reliability and validity of this method remain unclear [31, 32]. Therefore, it is necessary to consider both clinically examined dental treatment needs and the subjective oral health status to examine comprehensive unmet dental care needs. Although professionally assessed treatment needs (we operationalized and named it as normative needs to discern with subjective self-perceived oral health needs) represent a reliable and valid tool to examine dental needs, it may not represent true dental needs, in that normative dental treatment needs were perceived differently by individuals and might not always be realized to dental demands.

The objective of this study was to find disparities in unmet dental needs according to socioeconomic status (SES) given the consideration of both normative dental treatment needs and subjective oral health status among South Korean adults over the age of 19 years. Specifically, this study focused on two aspects: (1) to examine the inequality in unmet dental needs and (2) to explore the influence of socioeconomic factors on unmet dental care needs.

## Methods

### Data source and study population

This study used data of 2007–2009 Korean National Health and Nutrition Examination Survey (KNHANES IV), a cross-sectional and nationally representative survey conducted by the Korea Centers for Disease Control and Prevention (KCDC). KNHANES has been performed periodically since 1998 to investigate the health and nutritional status of Koreans. From the first (1998) to the third (2005) surveys, the KNHANES was carried out at 3-year intervals, but since 2007, it has been annually surveyed. KNHANES used a rolling sampling design with multi-stage clustered probability among the noninstitutionalized civilian population of South Korea (https://knhanes.cdc.go.kr/knhanes/eng/sub02/sub02_01.do). KNHANE IV 2007–2009 provided 3-year combined weights as well as weight of each year survey. The survey was composed of a health interview, a nutrition survey, and a health examination. Oral health has been included as part of the survey since the first year of KNHANES IV [33]. All participants in the survey participated voluntarily and provided informed consent.

The total number of adult participants in KNHANES IV was 18,406, and we included 17,141 adults aged 19 or older who participated in both the Health Interview Survey and the Oral Health Examination Survey. We excluded 1265 participants who did not respond to the question about unmet dental care needs. This study used pooled three annual surveys of KNHANES IV 2007–2009 to analyze data from 17,141 adults aged 19 years or older (weighted sample, 37,425,050).

### Oral examination

The KNHANES oral health examination was performed by trained public dentists at a mobile examination center (MEC). The health interview and oral health examination surveys were conducted over 3 days for each primary sampling unit (PSU) at the MEC, which traveled to locations across the country. The dental examinations were conducted by calibrated dentists with a light, mouth mirror, and periodontal probe with the subject seated on a dental chair. Dentist calibration training was carried out annually, and the results were compared with those of a reference dentist.

Dental caries in the permanent dentition was examined according to the WHO criteria [34]. During the oral examination, periodontal status was assessed using the community periodontal index (CPI) [35]. The five CPI scores used to evaluate periodontal health status were as follows: normal (CPI 0), gingival bleeding (CPI 1), calculus (CPI 2), shallow periodontal pockets of 4–5 mm (CPI 3), and deep periodontal pockets of 6 mm or greater (CPI 4). The highest resulting score was recorded as the CPI score for each individual. Groups were categorized according to periodontal status; non-periodontitis (CPI 0 - CPI 2 including normal and gingivitis) versus periodontitis (CPI 3 or CPI 4).

### Dependent variable

The dependent variable, the experience of unmet dental care needs, was defined as cases where participants answered “Yes” to the following KNHANES interview question: “Did you need dental care but were unable to receive dental treatment within the past year?”

### Independent variables

Based on the previous studies [27–30, 36], we included following variables as the independent variables for the analysis: demographic factors, socioeconomic factors, dental need factors, which were composed of both normative dental treatment needs and self-perceived oral status, and oral health-related factors. Sex, age, and marital status were included in the demographic variables, and education level, employment status, and income level were used as proxy variables representing SES. Income level was calculated by adjusting the monthly household income by the number of household members and was then categorized into the bottom 25% (1st quartile), 25–50% (2nd quartile), 50–75% (3rd quartile), and top 25% (4th quartile).

For the variable regarding dental needs, we included normative dental treatment needs and self-perceived oral health needs simultaneously. Normative dental treatment needs was confirmed by a dentist through an oral examination. During the oral examination in each survey, all 28 teeth were examined excluding the third molars to determine whether there was a need for treatment of one tooth surface or two or more tooth surfaces, artificial crown repair, restoration or pulp treatment, extraction, or other treatments. These were categorized into dichotomous treatment needed or treatment not needed. For self-perceived oral health needs, survey asked oral health status to respondents using five points Likert scale. We categorized it into three levels: good (excellent and good), fair (fair), and poor (poor and very poor).

The oral health-related variables included the number of decayed teeth, the presence of periodontitis, and the number of missing teeth. The number of decayed teeth included those currently existing in the maxilla and mandible, not including filled or missing teeth, and the number of missing teeth was identified from 28 teeth, excluding the third molars.

### Statistical analysis

Descriptive and bivariate analyses were performed on the general characteristics of the study subjects and the associated explanatory variables on unmet dental needs. We compared the differences in general characteristics between the two groups according to the presence of unmet dental needs. All the variables that were independently associated with the outcome in the bivariate analysis, with *p*-value less than 0.05 were fitted in the regression model. Multiple logistic regression analysis was performed using demographic, socioeconomic, normative dental treatment needs, subjective oral health needs, and oral health-related factors as explanatory variables. We examined multicollinearity and found that most were not highly correlated with one another. One exception was the number of decayed teeth, which was correlated with normative dental treatment needs. Since both were key indicators for our analyses, and the exclusions of one of those variables were virtually unchanged across the results, we included both variables in our final model.

All of the statistical analyses were performed with SAS software, version 9.2 (SAS Institute, Cary, NC), and applied sample design and sample weights to estimate the adjusted odds ratios (OR) and their 95% confidence intervals (CI) and significance levels.

## Results

An estimated 43.9% of South Korean adults had unmet dental care needs in the fourth KNHANES, with 7517 of 17,141 responding that they had perceived dental treatment needs but had been unable to receive treatment within the past year. The bivariate analysis for the association between the experience of unmet dental care needs and other independent variables is presented in Table 1. All of the independent variables showed significant associations with the dependent variable. The overall percentage of people with perceived unmet dental needs was greater in females; young adults; separated, divorced, or widowed people; those with low incomes; and in those with dental treatment needs, periodontal disease, caries, and poor oral health status. The high income group had a smaller percentage of people with unmet dental needs.

**Table 1:** Distribution of population characteristics and bivariate analysis of perceived unmet dental care needs

Variables	Experience of unmet dental care needs (% or mean ± SE)	No experience of unmet dental care needs (% or mean ± SE)	*P* value
Total (*n* = 17,141)	43.9 (*n* = 7517)	56.1 (*n* = 9624)	
Demographic factors			
Sex			<0.0001
Male (*n* = 7261)	40.8	59.2	
Female (*n* = 9880)	47	53	
Age (years)			<0.0001
19-39 (*n* = 5744)	46.2	53.8	
40-64 (*n* = 7553)	44.2	55.8	
65 or older (*n* = 3844)	35.9	64.1	
Marital status			0.0396
Married (*n* = 12,201)	43.9	56.1	
Separated/divorced/widowed (*n* = 2537)	46.9	53.1	
Single (*n* = 2312)	42.4	57.6	
Socioeconomic factors			
Education level			0.0121
University or higher (*n* = 4351)	43.4	56.6	
High school (*n* = 5882)	44.6	55.4	
Middle school (*n* = 1909)	46	54	
Elementary or lower (*n* = 4965)	42.5	57.5	
Employment status			0.0220
Employed (*n* = 9866)	44.6	55.4	
Unemployed (*n* = 7159)	42.9	57.1	
Income quartile			<0.0001
4^th^ quartile: High (*n* = 4180)	37	63	
3^rd^ quartile: Mid-high (*n* = 4195)	42.7	57.3	
2^nd^ quartile: Mid-low (*n* = 4156)	47.7	52.3	
1^st^ quartile: Low (*n* = 4181)	48.8	51.2	
Dental need factors			
Normative treatment needs			<0.0001
No treatment needs (*n* = 10,739)	36.1	63.9	
Treatment needs (*n* = 6018)	57.1	42.9	
Self-perceived oral status			<0.0001
Good (*n* = 2053)	19.7	80.3	
Fair (*n* = 6528)	34.8	65.2	
Poor (*n* = 8271)	57.7	42.3	
Oral health-related factors			
Number of decayed teeth (*n* = 18,406)	1.24 ± 0.03	0.58 ± 0.02	<0.0001^a^
Periodontal disease			<0.0001
No (*n* = 10,570)	42.1	57.9	
Yes (*n* = 5425)	48.7	51.3	
Number of missing teeth (*n* = 18,406)	3.02 ± 0.09	3.41 ± 0.09	<0.0001^a^

The total number of answers in subgroups does not equal the total number of subjects in this study, because subjects with missing answers were excluded from the analysis

Individual sample weights and the complex sample design including stratification and primary sampling units were considered in the analysis

*SE*, standard error

Significance set at *P* < 0.05. All *P* values except that for mean number of decayed teeth and missing teeth derived from chi-square test

^a^ Derived from *t*-test

The reasons for experiencing unmet dental care needs are presented in Table 2. The most common explanation was “economic reasons” at 41.4%, followed by “cannot leave the workplace or school” at 20.9%.

**Table 2:** Reason for experiencing unmet dental care needs (*n* = 7497)

Reason for experiencing unmet dental care needs	%
Economic reasons	41.4
Cannot leave the workplace or school	20.9
It is less important than other problems	14.5
Scared to receive dental treatment	10.9
Other	7
No one to babysit	3.1
Dental hospital is too far away	1.3
Difficulty moving or health problems	0.8

Table 2 includes only those who experienced unmet dental needs

Individual sample weights and the complex sample design including stratification and primary sampling units were considered in the analysis

All the independent variables with *p*-values less than 0.05 were selected for the multiple logistic regression analysis. Table 3 shows that the disparities in unmet dental care needs were significantly associated with income level, normative dental treatment needs, and self-perceived oral health status. Females were 1.45 -times more likely to have unmet dental needs than males, and separated, divorced, or widowed people were 1.15 -times more likely to have unmet dental needs than married people. Those with low incomes were approximately 1.4 -times more likely to have unmet dental needs than those in the highest income group. The highest levels of unmet dental care needs were observed in subjects who perceived their oral health status as poor and had needs for dental treatment that had been clinically confirmed by a dentist. Those who normative needed treatment and had periodontal disease were 1.61 - and 1.18 -times more likely, respectively, to have unmet dental needs than those who did not. Those who perceived their oral health as poor were 4.85 -times more likely to have unmet dental needs than those who perceived their oral health to be good. In contrast, people 65 or older and those who were unemployed were less likely to have perceived unmet dental needs.

**Table 3:** Logistic regression analysis results for factors related to perceived unmet dental care needs

Variables	Unmet dental care needs OR (95% CI)	Unmet dental care needs due to economic reasons OR (95% CI)
Total	*n* = 17,141	*n* = 7497
Demographic factors		
Sex		
Male	1.00	1.00
Female	1.45 (1.32–1.59)***	1.23 (1.07–1.41)**
Age (years)		
19–39	1.00	1.00
40–64	0.85 (0.75–0.95)**	1.51 (1.29–1.78)***
65 or older	0.59 (0.49–0.72)***	2.00 (1.52–2.62)***
Marital status		
Married	1.00	1.00
Separated/divorced/widowed	1.15 (1.01–1.32)*	1.47 (1.19–1.81)**
Single	0.81 (0.71–0.93)**	0.99 (0.81–1.22)
Socioeconomic factors		
Education level		
University or higher	1.00	1.00
High school	0.90 (0.81–0.99)*	1.31 (1.10–1.55)**
Middle school	0.96 (0.82–1.12)	1.74 (1.39–2.19)***
Elementary or lower	0.87 (0.75–1.00)	1.86 (1.50–2.29)***
Employment status		
Employed	1.00	1.00
Unemployed	0.90 (0.82–0.98)*	1.13 (0.98–1.29)*
Income quartile		
4^th^ quartile: High	1.00	1.00
3^rd^ quartile: Mid-high	1.19 (1.07–1.33)**	1.66 (1.36–2.03)***
2^nd^ quartile: Mid-low	1.39 (1.25–1.56)***	2.12 (1.75–2.57)***
1^st^ quartile: Low	1.42 (1.26–1.60)***	3.00 (2.44–3.69)***
Dental need factors		
Normative treatment needs		
No treatment needs	1.00	1.00
Treatment needs	1.61 (1.45–1.79)***	1.07 (0.92–1.24)
Self-perceived oral status		
Good	1.00	1.00
Fair	2.06 (1.78–2.37)***	1.14 (0.86–1.50)
Poor	4.85 (4.21–5.59)***	1.43 (1.10–1.86)**
Oral health-related factors		
Number of decayed teeth	1.11 (1.08–1.15)***	1.03 (1.00–1.07)
Periodontal disease		
No	1.00	1.00
Yes	1.18 (1.07–1.29)**	1.03 (0.90–1.17)
Number of missing teeth	0.99 (0.99–1.00)*	1.05 (1.03–1.06)***

Individual sample weights and the complex sample design including stratification and primary sampling units were considered in the analysis

*OR* odds ratio; *CI* confidence interval

***P < 0.001; **P < 0.01; *P < 0.05

Additionally, Table 3 shows that perceived unmet dental care needs due to economic reasons were saliently associated with income and education levels. The odds of having unmet dental needs due to economic reasons ware greater in females; older adults; and separated, divorced, or widowed people. The lowest income group was approximately 3 -times more likely to have unmet dental needs than the highest income group. Those with an elementary education or lower were 1.86 -times more likely to have unmet dental needs than those with a university education or higher. People with missing teeth and those with poor oral health status were more likely to have unmet dental needs.

## Discussion

This study attempts to analyze the association between inequality in received necessary dental care due to economic reasons and socioeconomic factors and whether this association may explain socioeconomic inequality in dental care needs. Among all of the factors related to unmet dental care needs, income level appears to be the most significant of the socioeconomic factors; thus, the possibility of experiencing unmet dental care needs increases as income level decreases. This result is consistent with those of Kim et al. [26], who showed that income was the most significant factor underlying the need for prosthetics for missing teeth.

The possibility of not receiving necessary dental treatment due to financial difficulties appeared to be higher when income and education levels were lower; thus, inequality in unmet dental care needs and socioeconomic factors were found to be strongly associated. A study by Lundegren [28] reported that the most important factor in predicting self-perceived oral treatment needs was a low education level, and Jang et al. [30] stated that the likelihood of having an unmet dental need increased when individuals had less than a high school education. Additionally, Maharani [37] found a high degree of unmet dental care needs among lower SES groups.

The results of this study showed that economic reasons were the most common causes of experiencing unmet dental care needs at 41.4%, consistent with previous studies [20, 30, 36, 38]. The results of our study of the reasons for unmet dental care needs showed that an inability to leave the workplace was the second highest cause after financial difficulties; consequently, it is evident that time constraints and financial difficulties are the most important reasons for unmet dental care needs. Unusually, unemployed individuals and those aged 65 or older had less unmet dental care needs than did the employed and young age groups in this study. Although it is difficult to present clear evidence about the result, we considered the several possibilities, and one of possible explanation is that the former groups have a higher personal threshold for dental treatments than they do for other types of consumption, such as subsistence-related commodities and treatments.

This study showed that normative treatment needs were relatively lower than perceived oral health status. However, this finding was inconsistent with those of Walter et al. [39], who showed that subjective needs were considerably lower than normative prosthetic treatment needs. There was also a discrepancy between the patient’s perception and the dentist’s decision regarding dental treatment needs in several studies [31, 39–43]. Table 1 is comparable in this respect. Because 36.1% of people who did not have clinical treatment needs reported that they had perceived unmet needs, it appears that the patients were well aware of the information concerning the need for treatment. Self-reported oral health is substantially influenced by personal views and usually differs from the clinically determined treatment needs. Perceived oral health needs are based on the individual’s perception of dental illness and vary from one individual to another according to sociocultural background, environmental factors, and SES. These factors can lead to differences in the reporting of the same objective oral health status [44]. Therefore, the patient’s subjective desire may be quantified, regardless of the objective dental treatment needs when the unmet need is measured focusing on subjective oral health needs. In contrast, 42.9% of people who had clinical treatment needs reported no perceived unmet needs in our study (Table 1), because objective need comes from the dentist’s assessment through identifying the signs of disease at an early stage when no symptoms of oral disease in terms of personal threshold have yet been observed [45]. Clinical treatment needs by the examining dentist may not be dental care needs in the subjective aspects according to the patient’s SES, severity of dental disease, and perception of dental illness. Relying on clinical diagnosis alone, without integrating the psychosocial dimensions of oral health need, may result in dramatic overestimates [46]. This study has demonstrated that unmet dental needs can be overestimated or underestimated under a specific point of view.

The overall improvements in oral health in the population have not changed the association between socioeconomic conditions and oral health inequalities. Indeed, oral health inequalities have persisted and even widened. Lisboa et al. [25] observed that socioeconomic status affects the curable dental needs of underprivileged Brazilian children and asserted that dental health programs should reduce inequalities in oral health status and access to oral health services for vulnerable populations. Watt [47] also emphasized that action is needed to make appropriate and effective dental treatment accessible to disadvantaged groups in society whose quality of life is most likely to be significantly influenced by oral diseases. In a study among adults in Sweden by Wamala et al. [48], insufficient access to dental care services played a critical role in worse oral health among socially marginalized individual. Public health interventions at the national level are therefore urgently needed to increase the accessibility of dental care services.

This study has some limitations. First, the design of the study was cross-sectional; as a result, there were difficulties inferring the causal effects, and our findings were limited to identifying associations. Second, the KNHANES dental examination data on treatment needs also had some limitations. Only the type of dental treatment need was identified in the KNHANES oral health examination. Therefore, further research is required to consider the severity or type of normative dental treatment needs among disadvantaged populations in the future.

Despite the above limitations, this paper contributes to the study of inequality of Korea in several ways. First, our findings can provide evidence of inequality in unmet dental care needs using nationally representative data of Korean adults. There are few reports of the current extent of inequality in unmet dental needs according to socioeconomic status (SES), although the Korean NHI aims to cover most of needed dental services. Second, oral examinations by dentists reflected normative needs for dental treatment, and objective oral health indicators, such as decayed teeth, periodontal disease, and missing teeth, were included in our analysis. Despite some technical difficulty of normative needs, normative dental needs have significant values, especially for those who do not response with any explicit dental needs but have some dental problems. This information is one of the benefits of oral examinations and cannot be gleaned from questionnaire surveys alone. Third, this study provides valuable information on a great number of unmet needs in Korea and can help policymakers both understand the distribution of population needs and determine priorities for the most effective allocation of limited resources to promote better oral health among socially disadvantaged populations.

## Conclusions

This study highlights inequalities in perceived unmet dental care needs according to socioeconomic factors, normative dental treatment needs, and poor oral status among South Korean adults. These results indicate that there are considerable inequalities in oral health status and that disadvantaged populations often face obstacles when accessing dental care services. Therefore, public health policies with the expansion of dental insurance coverage are needed to reduce such inequalities in unmet dental care needs and increase the accessibility of dental care services to vulnerable groups who are experiencing unmet dental care needs due to socioeconomic factors despite having normative and self-perceived needs for dental treatment.

## Abbreviations

CI: Confidence intervals
CPI: Community periodontal index
KCDC: Korea Centers for Disease Control and Prevention
KNHANES: Korean National Health and Nutrition Examination Survey
MECs: Mobile examination centers
NHI: National Health Insurance
OR: Odds ratios
PSU: Primary sampling unit
SES: Socioeconomic status
WHO: World Health Organization
